# Antifungal application of biosynthesized selenium nanoparticles with pomegranate peels and nanochitosan as edible coatings for citrus green mold protection

**DOI:** 10.1186/s12951-022-01393-x

**Published:** 2022-04-07

**Authors:** Mohamed F. Salem, Wessam A. Abd-Elraoof, Ahmed A. Tayel, Fahad M. Alzuaibr, Osama M. Abonama

**Affiliations:** 1 Department of Environmental Biotechnology, Genetic Engineering and Biotechnology Research Institute, University of Sadat City, Sadat City, 22857 Egypt; 2 Department of Fish Processing and Biotechnology, Faculty of Aquatic and Fisheries Sciences, Kafrelsheikh University, Kafr El Sheikh city, 33516 Egypt; 3 Department of Biology, Faculty of Science, University of Tabuk, Tabuk, 71491 Saudi Arabia; 4 Department of Industrial Biotechnology, Genetic Engineering and Biotechnology Research Institute, University of Sadat City, Sadat City, 22857 Egypt

**Keywords:** Characterization, Green synthesis, Mode of action, Nanopolymers coating, Nanocomposites, Orange, *Penicillium digitatum* infection

## Abstract

**Background:**

Citrus production and trading are seriously affected by fungal decays worldwide; the green mold infection by *Penicillium digitatum* could be the most disastrous. The substitutions of chemical and synthetic fungicides with effectual natural alternatives are global demands; plant extract from pomegranates peels (PPE), biosynthesized selenium nanoparticles with PPE (PPE/SeNPs) and chitosan nanoparticles (NCT) were suggested as efficacious fungicidal agents/nanocomposites to control *P. digitatum* strains.

**Method:**

PPE from *Punica granatum* was extracted and employed directly for synthesizing SeNPs, whereas NCT was produced using ionic gelation method of chitosan extracted from white prawn (*Fenneropenaeus indicus*) shells. The physiochemical, biochemical and structural characterization of generated molecules were conducted using infra-red spectroscopy, particles’ size (Ps) and charge assessment and electron microscopes imaging. Antifungal potentialities were investigated in vitro and in infected fruits with *P. digitatum* by applying NCT nanocomposites-based edible coating.

**Results:**

The synthesis of PPE-synthesized SeNPs and NCT was successfully achieved, the molecular bonding in synthesized agents/composites were proved with infrared spectroscopy to have both biochemical and physical interactions. The nanoparticles had 82.72, 9.41 and 85.17 nm mean diameters for NCT, PPE/SeNPs and NCT/PPE/SeNPs nanocomposites, respectively. The nanoparticles had homogenous spherical shapes and good distribution attributes. The entire agents/nanocomposites exhibited potent fungicidal potentialities toward *P. digitatum* isolates; NCT/PPE/SeNPs nanocomposite was the most forceful and significantly exceeded the fungicidal action of standard fungicide. The direct treatment of fungal mycelia with NCT/PPE/SeNPs nanocomposite led to remarkable lysis and deformations of *P. digitatum* hyphae within 12 h of treatment. The coating of infected orange with NCT-based edible coatings reduced the green mold infection signs by 91.7, 95.4 and 100%, for NCT, NCT/PPE and NCT/PPE/SeNPs based coating solutions, respectively.

**Conclusions:**

NCT, PPE-synthesized SeNPs, and their innovative nanocomposites NCT/PPE/SeNPs are convincingly recommended for formulating effectual antifungal and edible coatings to eliminate postharvest fungal pathogen, both with protection from their invasion or with destructing their existing infections.

**Graphical Abstract:**

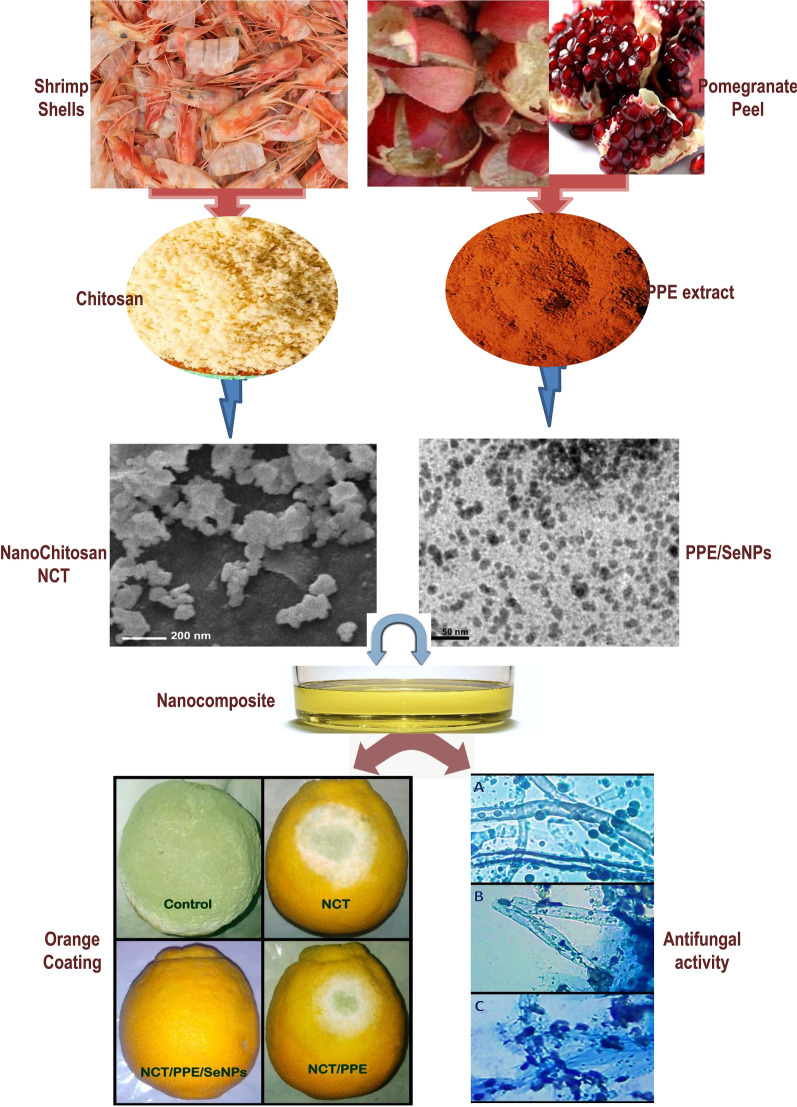

## Introduction

Citrus includes numerous varieties of important fruits that are susceptible to microbial damage by phyto-pathogens throughout planting, harvesting and commercializing, due to their prominent nutritional composition and elevated water content [1]. Citrus nature is typically acidic (pH ~ 2.2–4.0), which derive fungi to be the main responsible for most infections/deteriorations of these fruits [2]. The fungal contamination/invasion can occur in any step of fruits’ life. *Penicillium digitatum* is the necrotrophic fungus pathogen responsible for green mold that causes the foremost postharvest rot in citrus fruits; with massive economic losses reaching ≥ 90% from total postharvest damage in infected fruits worldwide [2, 3]. For example, although Egypt is one of the leading citrus producers globally, its worm climate can facilitate fungal pathogens growth, which seriously decreases the exported amounts from these crops [4].

Due to restraint regulation, potential carcinogenicity and acute toxicity, elongated degradation periods, environmental consequences, emerging fungal resistance, and intensified public concerns about chemical residues in crops, the uses of synthetic/chemical fungicides became increasingly worried [1, 5]. Consequently, the natural products usages and biological control approaches (including antagonistic microorganisms, bioactive natural derivatives and nano-biomaterials) attained great attentions as safe, effectual and environment-friendly alternatives to manage postharvest fungal infections with diminished risks for human and environment [5–7].

Nanotechnology and nanoparticles (NPs) are currently employed in most human-related fields; including biomedical, chemical, nutritional, biological, optical, mechanical, environmental and agricultural applications [8, 9]. The NPs are widely involved in the production, processing and preservation of human food, beginning from agricultural production and fertilization to presentation of foodstuffs to consumers [10, 11]. Due to NPs unique traits, e.g. enlarged surface area, very reduced size, high penetrability, distributions and shapes, they exhibit exclusively novel and enhanced characteristics over bulk particles. The customary used protocols for NPs synthesis, i.e. using chemical and physical procedures, could instigate numerous drawbacks. Physical methods require excessive energy supply, elevated costs, and produce limited NPs yield, whereas chemical procedures frequently have serious ecological and toxicological consequences [12]. The use of biomaterials like microbes, algae, biopolymers, plant materials, or their derivatives in the bio- (green) synthesis of NPs could efficaciously solve most of these foregoing drawbacks via providing facile, environment-friendly, cost-effective, controllable and high yield approaches [13, 14]. Plant (phyto) extracts and biochemicals were effectively utilized for metals NPs green synthesis; plentiful phytochemicals, e.g. phenolics, carotenoids, alkaloids, flavonoids, terpenoids, lignans, and further physiologically active molecules, were proved to generate and stabilize NPs with astonishing characteristics [11, 12].

The pomegranate (*Punica granatum* L.), are fruits that were recurrently mentioned in Holy Qur’an, Bible and Torah, and are cultivated worldwide for their taste and health benefits [15]. Because of their elevated contents from antioxidants, hydrolysable tannins, dyes, polyphenols and alkaloids, various parts of pomegranate plants are historically utilized for treating numerous ailments [16]. The extract of pomegranates peel (PPE) contains most of precious phytochemical components in pomegranate. PPE was documented as GRAS (Generally Recognized As Safe) and non-toxic even at high doses [2, 17]. Due to its outstanding potentialities (e.g. antioxidant, antibacterial, and antifungal properties), PPE was recurrently employed in food preservation and edible coating (EC) constitution to protect numerous crops and food stuffs from chemical and microbiological spoilages [18–20]. The PPE applications for biosynthesis of various metals NPs (e.g. silver, gold, zinc oxide and selenium) were successfully achieved, which validated the higher reducing, antioxidation and stabilizing capabilities of the extract [21–24]. These PPE-synthesized NPs have been experienced as potent antimicrobial, preservative and anticancerous agents.

Selenium (Se) trace element is a crucial component for both human and animal lives and functionality; ≥ 25 human seleno proteins/enzymes containing selenocysteine, which is essential for human health keeping [11]. The SeNPs have reduced cytotoxicity toward higher organisms (e.g. human, animals, fish, and crop plants) within permitted limits, but these NPs are highly bioactive to suppress bacteria, fungi, and eventually cancerous cells, which provide more applicability to SeNPs in biomedical, pharmaceutical and nutritional disciplines [24, 25]. The green synthesized SeNPs, especially with plant extracts, were actually incorporated in preservatives ECs for meat products and agricultural crops, antioxidant and anticancer pharmaceutical formulations [26–28].

Chitosan is the positively charged, linear and semi-crystalline biopolymer that derived from chitin [29, 30]. This astounding biopolymer has remarkable bioactive characteristics (including its biodegradability, eco-friendly, non-toxicity, bactericidal, wound healing, biosorption, and antioxidant potentialities); the chitosan could easily transfigured into emulsions, membranes, hydrogels, bandages, edible films and ECs [31–33]. The bioactivities, functionalities and formulability of this biopolymers are dramatically augmented by the transformation to chitosan nanoparticles (NCT), which are applicable for usage individually or as effectual carriers for further bioactive molecules in pharmaceuticals, food processing, biomedical, environmental, agricultural and nutritional sectors [30, 34, 35]. NCT was recurrently the nanopolymer of choice for fabricating bioactive ECs that protects foods and crops from microbial spoilage, quality and moisture loss, oxidation stress and pathogens invasion [35–37].

Accordingly, current investigation intended the production and synthesis of NCT and to apply PPE for biosynthesis of SeNPs, to make nanocomposites from these agents and evaluate their effectuality as *P. digitatum* fungicidal agents in vitro, and as ECs for controlling green mold in citrus fruit.

## Materials and methods

### PPE preparation

The fruit peels of pomegranate “*Punica granatum* L.”, organically farmed “at KFS research farm, Kafrelsheikh University, Egypt”, were manually attained after fruits washing and disinfection with 5% NaOCl solution for 5 min. Peels were rewashed with double-distilled water (DW) and worm air-dried (44 ± 2 °C for 62 h). After mechanical pulverization of dried peels, their powder (100 g, ~ 60 mesh size) was extracted with 1 L of diluted ethanol (70%), agitated in rotary shaker (IKA, KS 3000 I control, Germany) at 110×*g* for 65 h, at room temperature (RT; 25 ± 2 °C) and filtrated for discarding plant residues. The *P. granatum* peel extract (PPE) was dried at 41 °C under vacuum (Rotavap-R210, Büchi, Switzerland), and its attained powder was re-dissolved in DW to get 10% concentration.

### PPE-biosynthesis of selenium nanoparticles (SeNPs)

An aqueous solution (10 mM) from sodium selenite “Na_2_SeO_3_, Sigma-Aldrich, MO” was freshly prepared in DW. Subsequently, 10 ml from both PPE (1%, w/v) and Na_2_SeO_3_ solution (10 mM) were intermingled under stirring (AREX-6 magnetic stirer, VELP Scientifica Srl., Italy), at 610 × *g* for 55 min at RT. The eyesight vision of brownish-orange solution color signified PPE-biosynthesis of SeNPs. Centrifugation at 11.600 ×*g* for 37 min “SIGMA 2–16 KL centrifuge; GmbH, Germany” was performed to precipitate formed PPE/SeNPs matrix from solution. Portions from PPE/SeNPs matrix were subsequently washed (3 times) with DW then 2 times with ethanol (followed by centrifugation after each wash) to get plain SeNPs [26]. The PPE/SeNPs and plain SeNPs were then freeze-dried.

### Chitosan nanoparticles preparation and loading

The preparation and loading of NCT with PPE/SeNPs was adopted form formerly illustrated studies [18, 37]. The CT was produced from *Fenneropenaeus indicus* (white prawn) shells as previously demonstrated [27]. The extraction processes included shrimp shells’ drying, pulverization, deproteinization (in 2.0 N NaOH at RT for 250 min), demineralization (in 2.0 N HCl at RT for 250 min) and deacetylation (in 55% NaOH at 123 °C for 95 min). 5-Sodium tripolyphosphate “TPP; Sigma-Aldrich, MO” was utilized for NCT cross-linking; the dissolved chitosan (0.1%, w/v) in diluted acetic acid (1.5%, v/v) was the working solution and the TPP solution (0.5%, w/v in DW) was very slowly (rate 0.3 mL/min) dropped into the working solution wile stirred vigorously, until reaching 3.5: 1 ratio from chitosan: TPP solutions, respectively. The stirring (670 ×*g*) sustained for additional 75 min after TPP dropping and the formed NCT was gathered through centrifugation (11.250 ×*g* for 28 min). For nanocomposites formation from PPE/SeNPs and NCT (mentioned next as NCT/PPE/SeNPs), the PPE/SeNPs was added to CT solution (at 0.1% w/v), vortexed vigorously for 120 min before TPP dropping. Accordingly, the NCT was synthesized and uphold PPE/SeNPs in conjugation with the polymer nanoparticles (e.g. NCT/PPE/SeNPs nanocomposite), which was then centrifuged, washed with DW and lyophilized.

### Physiochemical and biochemical characterization

#### FTIR spectroscopic analysis

The produced compounds (PPE, NCT, PPE/SeNPs and their composites) were investigated spectrophotometerically for their infra-red spectra after mingling with KBr [e.g. intense powder mixing of samples with 1% (w/w) potassium bromide before analysis], operating FTIR “Fourier transform infrared spectroscopy, JASCO FT-IR-360, Tokyo, Japan”, with transmission mode and wavenumber range of 450–4000 cm^−1^.

#### Particles size and charging

The particles size (Ps) assessment of NCT, PPE-synthesized SeNPs, and their composites (PPE/SeNPs), along with NPs zeta (ζ) potentialities, were implemented through DLS “dynamic light scattering” system, operating zetasizer “Zeta plus, Brookhaven, USA”.

#### Nanoparticles’ ultrastructure

The SEM “JSM IT100, Scanning electron microscope, JEOL, Japan” was employed for NCT ultrastructure screening, including particles’ topography and dispersion, operating 20 kV accelerating voltage. The ultrastructure of PPE-synthesized SeNPs, i.e. their Ps, dispersion and shape, were further screened via TEM imaging “Leo 0430; transmission electron microscopy, Leica, Cambridge, UK”.

### Green mold isolates

The *Penicillium digitatum* isolates (e.g. *Pd* O, isolated from orange; *Pd* T, isolated from tangerine; and *Pd* S, standard strain ATCC-10030) were attained from green mold-infested fruits and identified in the EPCRS-KSU *“*Egyptian Phytomicrobial Collection and Preservation for Scientific Researches and Sustainability Development” Excellence Center*-* Kafrelsheikh University, Egypt. The identification of fungal isolates included their morphological patterns and was confirmed by MALDI-ToF MS analysis “Matrix-assisted laser desorption/ionization with time-of-flight mass spectrometry”. The fungal isolates were regularly propagated and screened using PDA and PDB media “Potato dextrose agar and broth, respectively; Oxoid, UK”, aerobically at 27 °C. The spores’ suspension (SS) was attained via soft scrapping of grown fungal cultures on PDA (7 days old) with sterile loop and washing the free spores with DW, vortexing this suspension well, and adjusting spores count to ~ 2 × 10^6^ spores/mL, using DW after counting spores with automatic cell counter “Countess-II FL, Thermo Fisher Scientific, MA”.

### In vitro evaluation of antifungal activity

The antifungal potentialities of NCT, PPE, PPE/SeNPs and NCT/PPE/SeNPs were in vitro assessed for inhibiting *P. digitatum*. Imazilil “Sigma-Aldrich, Taufkirchen, Germany” was used as standard fungicides for comparison; the fungicide was dissolved in 20% (v/v) DMSO “dimethyl-sulfoxide, Sigma-Aldrich, Germany”.

#### Well diffusion (WD) method

Agar WD method is broadly employed for assessing the antifungal potentiality, especially from natural derivatives [38]. The PDA plates were firstly inoculated and spread with 100 µL of fungal SS, then wells with 6 mm diameter were made using cork-borer and 50 µL (from 1% concentration of each compound in DW or imazilil in DMSO) were pipetted into wells. The plates were incubated under darkness for 72 h at 27 °C and the appeared inhibition zones (ZOI) around wells were determined in millimetre [39].

#### Minimum fungicidal concentration (MFC)

The MFC of each screened compound (NCT, PPE, PPE/SeNPs and NCT/PPE/SeNPs) or imazilil toward *P. digitatum* isolates, were appraised using diluted broth method [6, 40]. Gradual concentrations (10–100 mg/mL) from challenging compounds were intermingled with PDB and inoculated with isolates SS. The media were incubated aerobically for 8 days, then 100 µL from each trial were spread onto fresh PDA plates and incubated. The absence of any grown cells onto PDA plates after 7 days of incubation designated the MFC from each compound toward fungal isolates. Considering imazilil as the standard fungicide, the MFC ranges that distinguish susceptible and resistant *P. digitatum* isolates was set as follow: ≤ 25 mg/mL (highly susceptible), 25–50 mg/mL (moderately susceptible), 50–75 mg/mL (partially resistant) and > 75 mg/mL (resistant).

### Antifungal edible coating

#### Edible coating (EC) preparation

The ECs preparation was adopted with some modification from Tayel et al. [6]. Briefly, the bioactive NCT-based materials (e.g. NCT alone, NNCT/PPE and NCT/PPE/SeNPs) were dissolved in acidified DW (pH 5) at their MFCs values; glycerol was integrated to solutions as plasticizer with 5% v/v.

#### Fruits treatment

Organically farmed Navel oranges (*Citrus sinensis*) were gathered from the USC Research farm “University of Sadat City, Egypt”; the fruits average diameter was 8.3 ± 0.4 cm and they were all free of any surface damage, injuries or apparent infections. Before coating, fruits were showered with DW, disinfected via immersion in NaOCl solution (5%) for 2.5 min, rinsed again with DW and drained until dryness. Orange fruits were wounded at one point in the equator using sterile cutter (3 mm wide × 3 mm deep). Injured fruits were submerged in 1200 mL of *Pd* O fungal SS for 8 min (to generate artificial infections), drained for 15 min and aseptically air dried for 90 min. Fruits were subsequently dipped into NCT-based ECs (~ 1L) for 5 min with stirring and RT air-dried. The treated oranges with NCT-free EC represented the control group. ECs coated oranges were kept in sterile humid room (90% RH) for 14 days at controlled RT. The diameters of fungal lesions (LD) were gauged routinely within inoculation period [7].

#### Microscopic observation of treated fungal mycelia

The alteration in *P. digitatum* mycelial morphology, after treatment with MFC from NCT/PPE/SeNPs, was microscopically detected; with digital optical microscope “Labomed Lx400; Labo America Inc., Fremont, CA”, after incubation of fungal mycelia with the nanocomposite for 12 and 24 h under stirring and staining of treated mycelia with lactophenol-blue (Sigma-Aldrich, MO).

### Statistical analysis

Experiments were performed in triplicates; their means and SD (standard deviations) were computed and compared using SPSS V-20 software. The differences significance was computed by one-way ANOVA at *p* ≤ 0*.*05.

## Results

### Chitosan characteristics

The assessment of produced chitosan physiochemical physiognomies revealed that the biopolymer had a DD of 88.13%, 97.4% solubility in acidic pH solution (1.5% acetic acid aqueous solution), without external heating or sonication, and a 46.72 kDa molecular weight.

### Biochemical analysis of employed materials

**Fig. 1:**
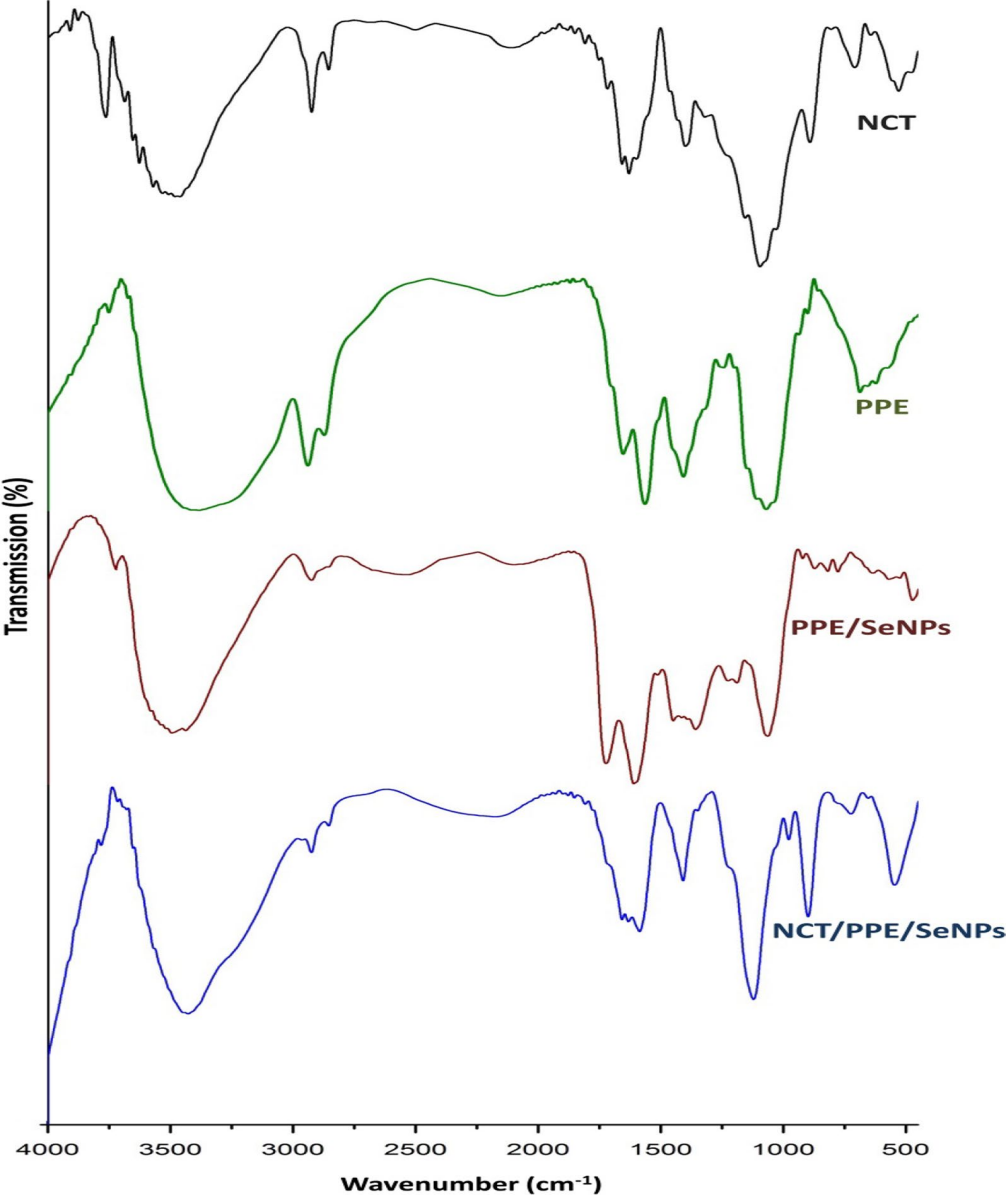
Infra-red spectra of employed materials, including nanochitosan (NCT), pomegranate peels (PPE) extract, PPE synthesized SeNPs (PPE/SeNPs) and their composites

The FTIR biochemical analysis of fabricated compounds are presented in Fig. 1. For the NCT spectrum (Fig. 1-NCT), the band around 3426 cm^−1^ corresponded to stretched O–H and N–H intramolecular hydrogen bonds. The main designative bands in NCT notably appeared at 2919, 2874, 1655, 1411, 1358, 1153, 1321, 1196, 1066 and 1025 cm^−1^.

The PPE spectrum designated the key biochemical bonds of the extract (Fig. 1-PPE); the designative peaks in PPE spectrum were detected at 3347, 2977, 2888, 1723, 1601, 1361, 1439, 1182, 1042 and at 879 cm^−1^.

The leading biochemical groups/bonds in PPE that contributed in SeNPs synthesis/reduction were screened from their combined FRIR spectrum (Fig. 1-PPE/SeNPs). Many distinctive bands in PPE spectrum have been shifted, disappeared or had altered intensities after interaction with SeNPs. Other bands were emerged, as indicators for novel formed bonds between PPE biomolecules and SeNPs.

The FTIR spectrum of NCT conjugates with PPE/SeNPs illustrated their strong interactions (Fig. 1-NCT/PPE/SeNPs), the spectrum had numerous characteristic peaks from each compound.

### Structural and physiochemical analysis of nanoparticles

The PPE phyto-reduction of sodium selenite (Na_2_SeO_3_) to SeNPs was attained and visually displayed from changing the color of synthesis solution from pale yellow to intense reddish-orange. The estimated Ps and ζ potential of NCT, PPE/SeNPs and NCT/PPE/SeNPs are illustrated in Table 1. The PPE could effectually reduce SeNPs to miniature particles with the mean Ps diameter of 9.41 nm. The PPE-phytosynthesized SeNPs exhibited strong negative ζ potential (− 31.4 mV), which could preserve NPs dispersion and prevent aggregation. For NCT, the nanopolymer mean Ps diameter was 82.72 nm, and these particles had highly positive charges (+ 38.8 mV). With conjugation of both synthesized NPs (NCT/PPE/SeNPs), the composite particles had slightly bigger Ps mean diameters and range, indicating SeNPs integrations and capping within NCT. Additionally, the nanocomposite had positive ζ potential (+ 31.7), which suggests that SeNPs became mostly embedded inside nanopolymer particles and the outer surface was from NCT.

**Table 1:** Ps distribution and ζ potential of fabricated nanochitosan (NCT), biosynthesized selenium nanoparticles with PPE (PPE/SeNPs), and their composites (NCT/PPE/SeNPs)

Nanoparticles	Size range (nm)	Mean diameter (nm)	Zeta potential (mV)
NCT	22.18–159.73	82.72	+ 38.8
PPE/SeNPs	3.41–21.35	9.41	− 31.4
NCT/ PPE/SeNPs	24.58–164.71	85.17	+ 31.7

**Fig. 2:**
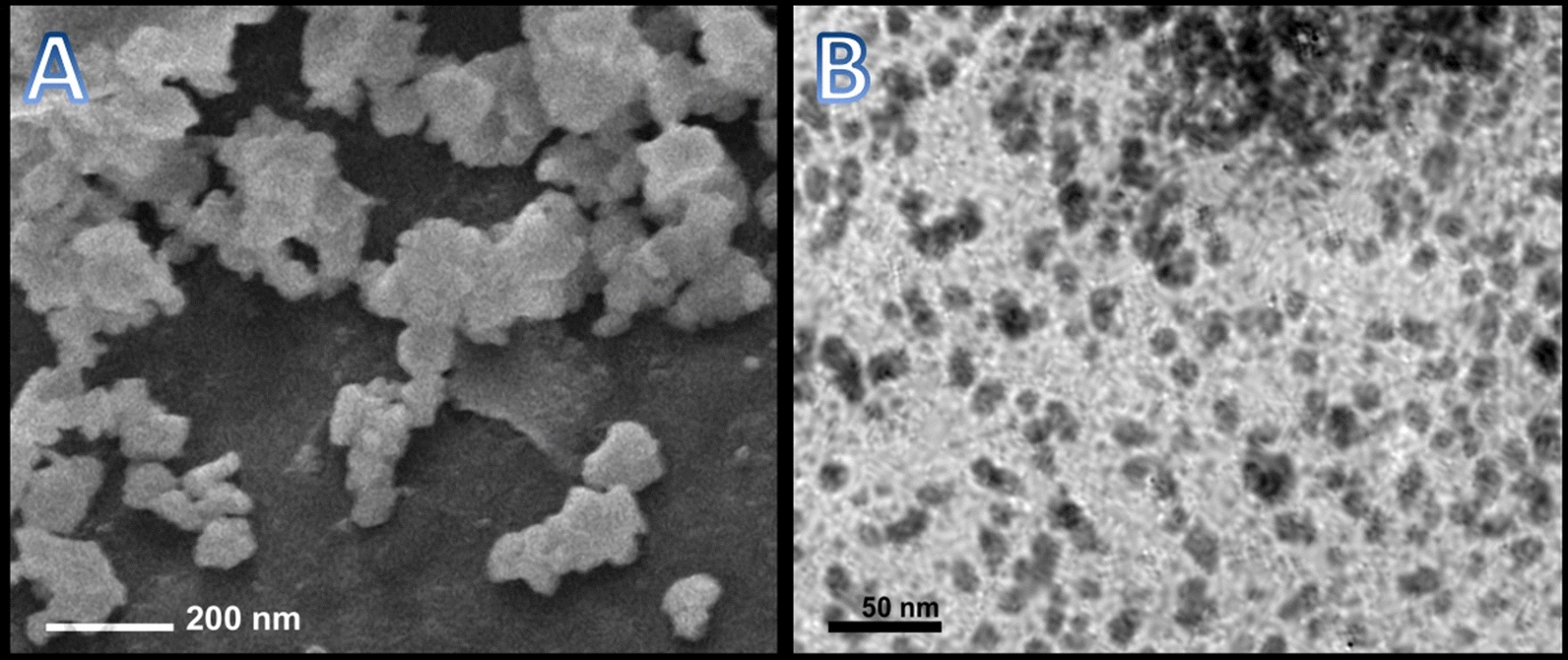
Nanoparticles’ ultrastructure using SEM for nanochitosan (**A**), and TEM for pomegranate peels-synthesized SeNPs (**B**)

The elevated ζ potential of synthesized nanoparticles/composites could effectually conserve their stability as evidenced from electron microscope images (Fig. 2).

The SEM imaging of NCT ultrastructure indicated their semispherical shapes and good distribution, with average Ps of ∼ 83.45 nm (Fig. 2A), which harmonized attained data from DLS analysis (Table 1).

The TEM topography of phytosynthesized SeNPs by PPE (Fig. 2B) designated the SeNPs diminished Ps and consistent distribution (with 4.07–20.94 nm size range and 9.54 nm mean diameter), matching the DLS results (Table 1). The nanometal particles had no apparent aggregation and appeared with spherical shapes and homogenous Ps (Fig. 2B).

### Antifungal activity of produced compounds

The in vitro antifungal assessments of experimented agents (NCT, PPE, PPE/SeNPs and NCT/PPE/SeNPs), compared to imazilil (standard fungicide), against *P. digitatum* isolates, are illustrated using qualitative and quantitative assaying methods (Table 2). The entire experimented agents/composites exhibited remarkable antifungal activities toward all *P. digitatum* isolates. The nanocomposite (NCT/PPE/SeNPs) was the strongest and their fungicidal activities significantly exceeded the other agents and the commercial fungicide imazilil actions. The antifungal synergism between agents was evidenced, as the combination of multiple agents (e.g. PPE/Se/NPs and NCT/PPE/SeNPs) displayed more forceful effects than individual compounds (e.g. NCT and PPE). Regarding *P. digitatum* isolates’ sensitivity to challenging agents, the isolate *Pd* O was the most resistant and the *Pd* 10,030 was the most sensitive, as evidenced from their ZOI and MFC values (Table 2). All fungal strains were “highly susceptible” to both PPE/SeNPs and NCT/PPE/SeNPs, while they were considered as “moderately susceptible” toward NCT and PPE.

**Table 2:** In vitro antifungal assessment of experimented agents against *Penicillium digitatum* isolates, via measurement of inhibition zones diameter (ZOI, mm) and minimal fungicidal concentrations (MFC, mg/mL)

Antifungal compound^1^	*Penicillium digitatum* isolates					
	*Pd* O		*Pd* T		*Pd* 10,030	
	ZOI^2^	MFC^3^	ZOI	MFC	ZOI	MFC
NCT	18.3 ± 1.5^a^	35.0	19.1 ± 1.8^a^	32.5	20.4 ± 1.3^a^	32.5
PPE	21.6 ± 1.7^b^	32.0	21.8 ± 2.1^b^	30.0	22.2 ± 2.3^b^	27.5
PPE/SeNPs	25.3 ± 2.2^c^	25.0	25.8 ± 1.9^c^	22.5	25.5 ± 1.9^c^	25.0
NCT/PPE/SeNPs	30.1 ± 2.0^d^	17.5	31.4 ± 2.4^d^	15.0	30.8 ± 2.8^d^	12.5
Imazilil	23.7 ± 1.9^c^	27.5	24.1 ± 2.3^c^	25.0	25.3 ± 2.6^c^	22.5

^1^The experimented agents included nanochitosan (NCT), extract of pomegranate peels (PPE), biosynthesized selenium nanoparticles with PPE (PPE/SeNPs), and their composites, compared to standard fungicide imazilil

^2^Dissimilar superscript letters (a-d) in a column appointed significant difference at P > 0.05

^3^The MFC ranges for isolates susceptibility were set as: ≤ 25 mg/mL (highly susceptible), 25–50 mg/mL (moderately susceptible), 50–75 mg/mL (partially resistant) and > 75 mg/mL (resistant)

### Microscopic observation of treated fungal mycelia

**Fig. 3:**
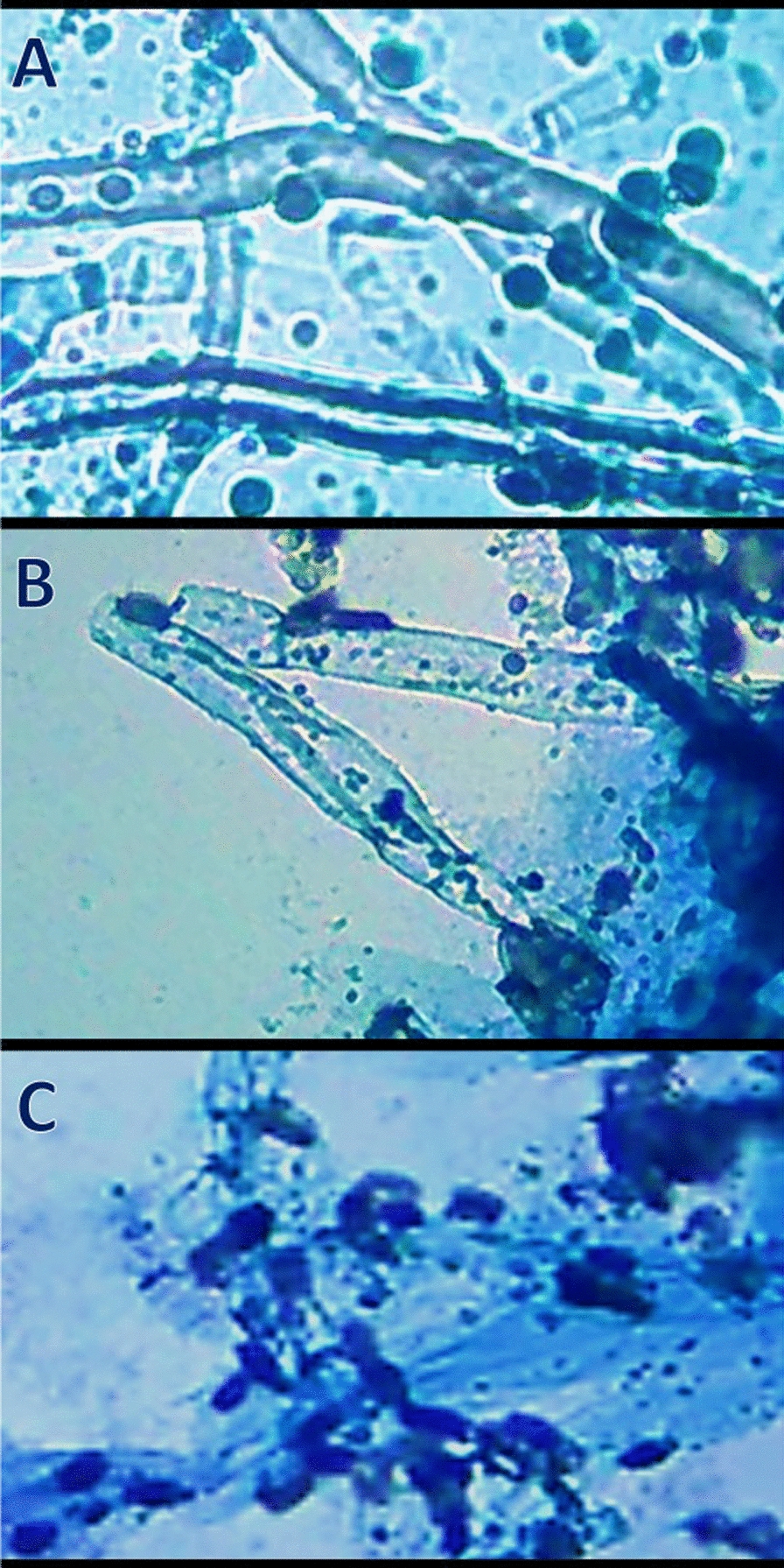
Optical microscope observations of treated *Penicillium digitatum* mycelia with composited nanochitosan/pomegranate peels extract/SeNPs for 6 h (**B**) and 12 h (**C**), compared to untreated mycelia (**A**)

The alteration in *P. digitatum* mycelial morphology, after treatment with MFC from NCT/PPE/SeNPs, was microscopically detected (Fig. 3). The fungal mycelium in the trial beginning appeared with healthy and strong feature; the mycelial wall/surface were smooth and dense with no apparent distortions (Fig. 3A). After 6 h of exposure, the mycelium had irregular swellings and fragmentations, with the softening of their walls and appearance of distortion signs (Fig. 3B). By the exposure end, after 12 h, the entire fungal mycelia were mostly lysed and lost their distinctive structures; the interior cellular components leaked outside the hyphae at this stage (Fig. 3C).

### Treatment of orange fruits with NCT-based edible coating

**Fig. 4:**
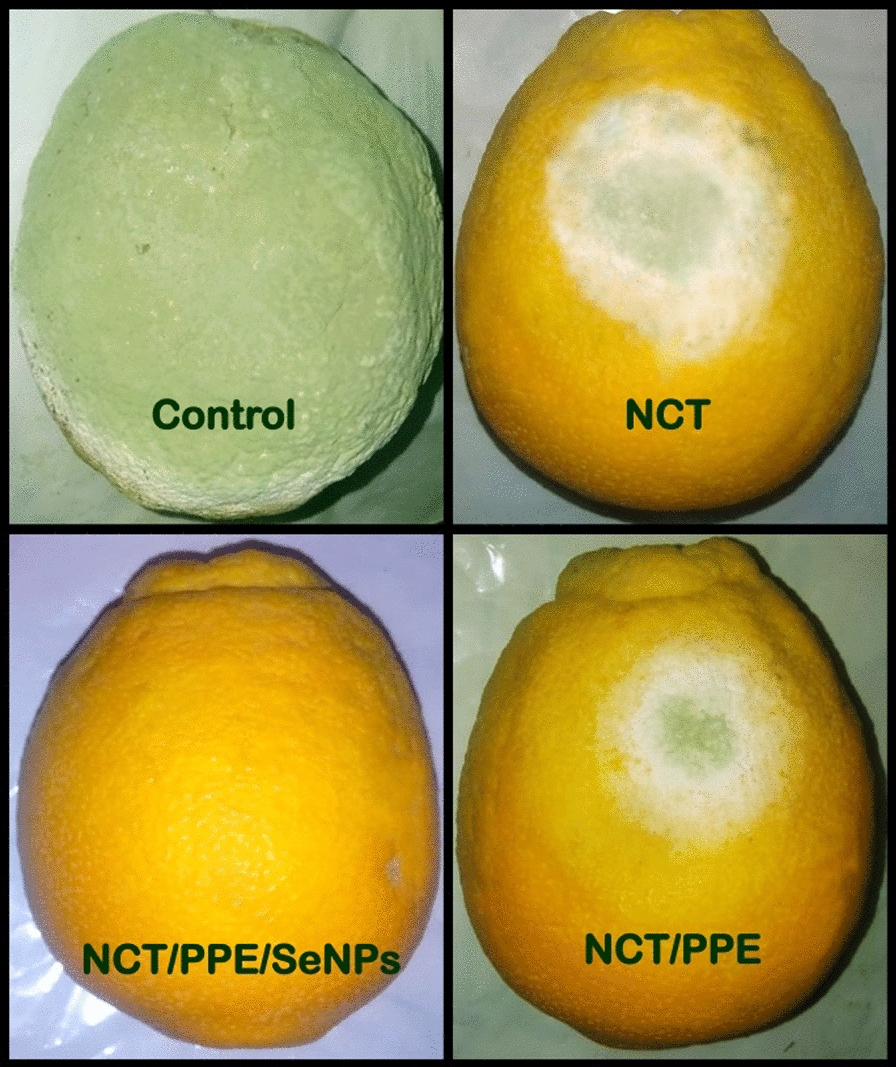
Consequences of orange fruits coating with formulated nanochitosan (NCT), NCT with extract of pomegranate peels (NCT/PPE) and NCT/PPE/SeNPs, after 10 days of infection with *Penicillium digitatum*

The consequences of orange fruits treatments with NCT-based edible coatings (i.e. plain NCT, NCT/PPE and NCT/PPE/SeNPs), after 10 days of infection with *P. digitatum* O, is photographically shown (Fig. 4). While the control fruits (uncoated) became fully decayed and the fungal growth covered the entire fruit, the coated fruits exhibited reduced infection signs (Fig. 4). The coating with NCT/PPE/SeNPs based solution could completely protected orange fruits from any infestation signs and preserved the fresh-like appearance and texture of treated fruits. The infection signs in NCT-coated fruits covered ~ 8.3 ± 1.2% from fruits’ surface area, whereas in NCT/PPE-coated fruits, the fungal infestation covered only ~ 4.6 ± 0.7% from fruits’ surface area (Fig. 4). Interestingly, the fruits’ coating with NCT/PPE/SeNPs nanocomposite could maintain the quality of coated fruits for further 20 days after the experiment duration, without any infection signs.

## Discussion

The chitosan was successfully generated in current study; the attained chitosan physiognomies suggested its successful extraction, as chitosan should have ≥ 70% DD, which indicated effectual deacetylation of chitin substrate [31, 37, 41].

The FTIR analysis indicated the most effectual bonds/groups in screened molecules. For the NCTspectrum (Fig. 1-NCT), it had the main characteristic bands of the typical bands of natural chitosan [41, 42]. The band around 3426 cm^−1^ indicated the main locations for TPP interactions with chitosan [37]. The bands appeared at 2919 and 2874 cm^−1^ are indicatives to C–H symmetric/asymmetric stretching, which are typical bands for polysaccharides. The following detected bands are distinctive to NCT biochemical bonding: ~ 1655 cm^−1^ (stretched C = O of amide I); 1321 cm^−1^ (vibrated C–N stretching); 1411 and 1358 cm^−1^ (CH_2_ bending and CH_3_ symmetrical deformations); 1153 cm^−1^ (bridge of C–O–C asymmetric stretching); 1066 and 1025 cm^−1^ (C–O stretching) [43–45]. The appeared peaks at 1153 and 1066 cm^−1^ indicated the C–O overlapping and formation of NCT after interaction of PO_4_ and NH_4_ groups in NCTmolecules; and also the peak at 1196 cm^−1^ that is corresponding to stretched P = O pond, validated NCTsynthesis following TPP interaction [37, 43].

The designated biochemical bonds in PPE spectrum included the bands at 3347 cm^−1^ [bonded –NH and –OH groups of carboxylic acid (CA), gallic acid, tannic acid and ellagic acid] [17, 46]; 2977 cm^−1^ [stretched C–H vibration of methyl and methoxy groups and stretched vibration of –CH_3_/ –CH_2_ groups of CA]; 2888 cm^−1^ [vibrated C‒H stretching of alkyl];1723 cm^−1^ [N–H bonds of carboxylic and amides groups]; 1601 cm^−1^ [stretched C = C vibration of aromatic rings and vibrated N–H of amines]; 1361 cm^−1^ [C–O stretching in acid groups]; 1439 cm^−1^ [aromatic rings]; 1182 cm^−1^ [–OH deformation and C–O stretching of primary alcohols]; 1042 cm^−1^ [–OH deformation and C–O stretching of tertiary alcohols]; and at 879 cm^−1^ [aromatic ring vibration] [21, 45–47].

The combined PPE/SeNPs spectral analysis indicated the most responsible groups in PPE for the biosynthesis of SeNPs. The PPE band at 3426 cm^−1^ shifted to 3482 cm^−1^ in PPE/SeNPs spectrum, indicating Se interaction with N–H and O–H groups, whereas the C–H band (at 2888 cm^−1^ in PPE spectrum) mostly disappeared in PPE/SeNPs spectrum, indicating its roles in SeNPs conjugation/reduction [22]. Also, the bands in PPE spectrum at 1723 cm^−1^ (N–H of amides and CA groups) and at 1601 cm^−1^ (aromatic rings C = C) were remarkably shifted, as indicator of their roles in SeNPs synthesis/reduction [23, 46]. The beak at 1439 cm^−1^ (aromatic rings in PPE spectrum) shifted to 1378 cm^−1^ in PPE/SeNPs spectrum. Furthermore, the emergence of multiple notable bands at 1603 cm^−1^ and in the range of 756–812 cm^−1^ in PPE/SeNPs spectrum clearly indicated the formation of novel bonds and vibrated bending (mainly of Se–O) after interactions of Se ions with PPE biomolecules [24, 48]. These detectable bands in PPE/SeNPs spectrum strongly validated the PPE potentiality for conjugating, reducing and stabilizing SeNPs; the reduction/stabilizing of SeNPs forms are predominantly depending on the biomolecules’ nature and their stabilization capability that enable Se ions interaction with them [24, 46, 49]. Thus, PPE could be advocated as valued stabilizer/reducer for SeNPs biosynthesis.

FTIR analyses are useful to assess whether the conjugation of PPE/SeNPs with NCT is physical or chemical entrapment; if minimal or no deviations from parental compounds FTIR spectra were observed, the physical entrapment is expected, whereas spectral bands’ shift or varied intensities indicate probable chemical interactions between molecules [20]. As many peaks in NCT/PPE/SeNPs spectrum were shifted and varied from their parental compounds (NCT and PPE/SeNPs), along with the identical peaks that were detected from both agents, the spectral comparison could strongly indicates both biochemical and physical interactions during PPE/SeNPs entrapment within NCT [20, 44].

The NCT high capability of capping SeNPs and formation of highly stable nanocomposites with minute Ps were demonstrated formerly [27, 42]. Generally, NPs with elevated ζ potentials (≥ + 30 mV or ≤  − 30 mV) display high stability and dispersity degrees due to electrostatic repulsion between particles [50, 51].

The NCT synthesis via TPP cross-linking was proven as effective operative protocol, employing ionic-gelation interaction; the synthesized NCT with such protocol had astonishing properties for practical employment either as plain bioactive molecules or as nanocarriers for other bioactive constituents, or bases for active ECs [27, 37].

The tiny Ps of phytosynthesized SeNPs and their remarkable dispersion indicate the advanced capability of PPE for reducing/stabilizing SeNPs. The antioxidating, radical scavenging and reducing potentialities of PPE were acknowledged, principally due to the extract contents from phenolics, tannins, alkaloids, and flavonoids (e.g., punicalagin, gallic tannins, catechins, quercetin, kaempferol, ellagic acid, catechol, castalagin,, gallocatechin, and granatin) [15, 23, 24, 48]; these precious phytocompounds could effectually play principal roles in SeNPs biosynthesis.

The general antimicrobial and specific antifungal potentialities of screened agents have been documented toward various microbial pathogens [3, 19, 36, 52]. The chitosan microbicidal actions depend mainly on its surface positive charges, which enable its attachment and interaction with microbial membranes and internal organelles, beside increase of intracellular ROS “reactive oxygen species” production, suppress cellular bioactivities and upsurge cellular membranes’ permeability [32, 33]. These actions become more forceful and effectual by transforming the biomolecule to nanoforms (e.g. NCT), because of the increased reacted surface area and tiny Ps that enable more effectual interactions and biocidal actions [30, 32]. The PPE antimicrobial activities are principally attributed to its bioactive phytoconstituents (e.g. phenolics, tannins, alkaloids, flavonoids and acids), which were previously investigated, validated and applied for controlling numerous bacterial and fungal pathogens [15, 17, 19].

The PPE mediated nanometals were also verified as potent microbicidal agents that have the synergistic actions from both PPE and synthesized nanometals, including Se, Ag, Au and Zn NPs [22, 23, 48].

For the NCT/PPE/SeNPs, which was innovatively composited in current investigation, the antifungal synergism between compositing agents (NCT, PPE and SeNPs) was clear and forceful, as evidenced from the widest ZOIs and least MFCs values; this indicates that composites ingredients could preserve their distinctive antifungal actions. Matching findings were recently reported [20], employing NCT and PPE composites as antioxidant conjugates. Additionally, the application of NCT for carrying, capping and delivering further bioactive molecules such as plant extracts, essential oils and nanometals have been reported to augment their combined actions as antimicrobial, antioxidant or even anticancerous nanocomposites [29, 43–45]. These former findings could verify the obtained role here of NCT to strengthen the antifungal actions of both PPE and SeNPs.

The antifungal potentialities of NCT and its parent chitosan have been proved toward numerous postharvest pathogens; the exact modes of action still vague, but it could be suggested that the positively charged NCT can attach hyphal walls, interact with fungal membranes and penetrate within these membranes to inhibit/destruct the fungal biosystems and lead to their lysis [30, 32, 34]. The PPE/SeNPs are suggested to damage microbial cells because of their combined biocidal activities. The destruction and deformation of *P. digitatum* hyphae was formerly observed, after treating them with PPE-related phytochemicals [13], which advocates current obtained results, in addition to SeNPs antifungal action.

The innovative nanocomposite here (NCT/PPE/SeNPs) is suggested to perform multiple actions; firstly the NCT carries/holds PPE/SeNPs to the fungal hypha and attaches/interacts with them to cause softening and partial lysis of membranes, then it could penetrate inside the hypha and the liberated PPE/SeNPs beside NCT are capable to intermingle with intracellular organelles/biosystems to suppress their vital functions, which consequently lead to fungal deformation and lysis [30, 32, 53].

The chitosan- and NCT-based ECs were recurrently validated as effectual treatments for preventing postharvest decays/losses in many agricultural crops. The main distinguished functions of these ECs, beside the antimicrobial actions, are to form barriers against fungal new infection, protect fruit from moisture loss and manage the respiration and over-ripening of coated crops [29, 30, 35, 36]. PPE was also the principal component of ECs for many fruits; the extract could eliminate microbial growth on fruit and maintain their freshness because of powerful PPE antimicrobial and antioxidant potentialities [3, 7]. Furthermore, the conjugation of chitosan and PPE in ECs of fruits and vegetables could have higher functionalities than each individual component for preserving coated crops, enlarging their shelf lives and prevent their microbial decays [18, 46]. These functions were suggested to be elevated with conjugation of NCT with PPE and their usage in ECs of fruits. NCT has higher capabilities to encase the whole fruits surface, fill their pores, deliver the accompanied molecules to fruit, and prevent them from fungal invasions and quality loss [20, 37, 45]. The combination of NCT with PPE/SeNPs is innovatively presented here to employ this nanocomposite as effectual EC for orange fruit; the biosafe nature of NCT and its elevated capping ability could provide more biosafety attributes toward the potential toxicity from SeNPs, as the embedding of nanometals into biopolymer matrix was previously proven to diminish their biotoxicity and increase their biocompatibility and safety [27, 31, 35, 42, 53].

## Data Availability

All data generated or analysed during this study are included in this published article.
